# A systematic review on the cardiovascular pharmacology of *Emblica officinalis * Gaertn.

**DOI:** 10.15171/jcvtr.2018.20

**Published:** 2018-09-25

**Authors:** Fataneh Hashem-Dabaghian, Mojtaba Ziaee, Samad Ghaffari, Farzaneh Nabati, Saeed Kianbakht

**Affiliations:** ^1^Research Institute for Islamic and Complementary Medicine, Iran University of Medical Sciences, Tehran, Iran; ^2^School of Persian Medicine, Iran University of Medical Sciences, Tehran, Iran.; ^3^Cardiovascular Research Center, Tabriz University of Medical Sciences, Tabriz, Iran; ^4^Medicinal Plants Research Center, Institute of Medicinal Plants, ACECR, Karaj, Iran

**Keywords:** *Emblica officinalis*, Cardiovascular Disease, Pharmacology, *Phyllanthus emblica*, Amla

## Abstract

***Introduction:*** The *Emblica officinalis* (EO) fruit has traditionally been considered as a cardioactive medication and has demonstrated remarkable cardiovascular effects in the pharmacological literature. The present study systematically reviews EO’s potential for prevention and therapy of cardiovascular diseases (CVD).

***Methods:*** PubMed, ScienceDirect, Scopus, Proquest, Ebsco, Google, Google Scholar, Ovid, and Cochrane databases were searched from 1966 to 2017 for the English and non-English literature using the terms including the cognates of EO including *amla, Emblic myrobalan, Emblica officinalis, Emblica pectinata *, Indian gooseberry, and *Phyllanthus emblica * together with antioxidant, arrhythmia, cardioprotective, cardiotoxicity, heart disease, heart failure, hyperlipidemia, hypertension, myocardial dysfunction, and oxidative stress. The inclusion criteria were in vitro, animal, and clinical cardiovascular pharmacological studies conducted on EO and full-text accessibility. The exclusion criterion was studies in which a combination of EO and at least one other plant was investigated. The reference lists of the retrieved articles were also searched manually for additional eligible articles. The methodological quality of clinical trials was assessed by the Jadad scale, and animal studies were evaluated by the ARRIVE checklist.

***Results:*** Nineteen articles concerning the cardiovascular pharmacological effects of EO were included in this review. The plant has shown antiatherogenic, anticoagulant, hypolipidemic, antihypertensive, antioxidant, antiplatelet, and vasodilatory effects as well as lipid deposition inhibitory properties. Moreover, it prevents from doxorubicin and isoproterenol cardiotoxicity and myocardial ischemia/reperfusion injury, and improves vascular endothelial function in animal studies. Some high-quality clinical studies report the vasodilatory and myocardial antioxidant properties as well as anti-platelet aggregation effects of this plant.

***Conclusion:*** EO influences various cardiovascular risk-factors. However, there is not sufficient evidence to confirm the plant efficacy in preventing and treating CVD.

## Introduction

Cardiovascular diseases (CVD) are the leading cause of death worldwide, resulting in 17.9 million deaths in 2015^1^ and expectedly exceeding 23.6 million by 2030.^2^

Some medicinal plants and food components (as monotherapy or adjunct to standard pharmacotherapy) have traditionally been used to treat CVD and have demonstrated various cardiovascular pharmacological effects.^3-6^ *Emblica officinalis* is one of the medicinal plants whose cardiovascular effects have been considered both in the traditional medicine and in the modern scientific literature.

**Figure 1:**
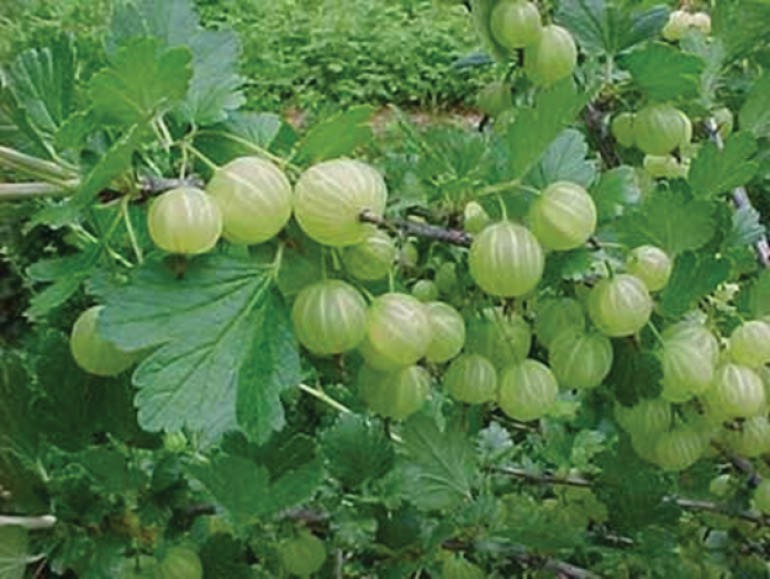
Picture of the *Emblica officinalis* fruits.

**Figure 2:**
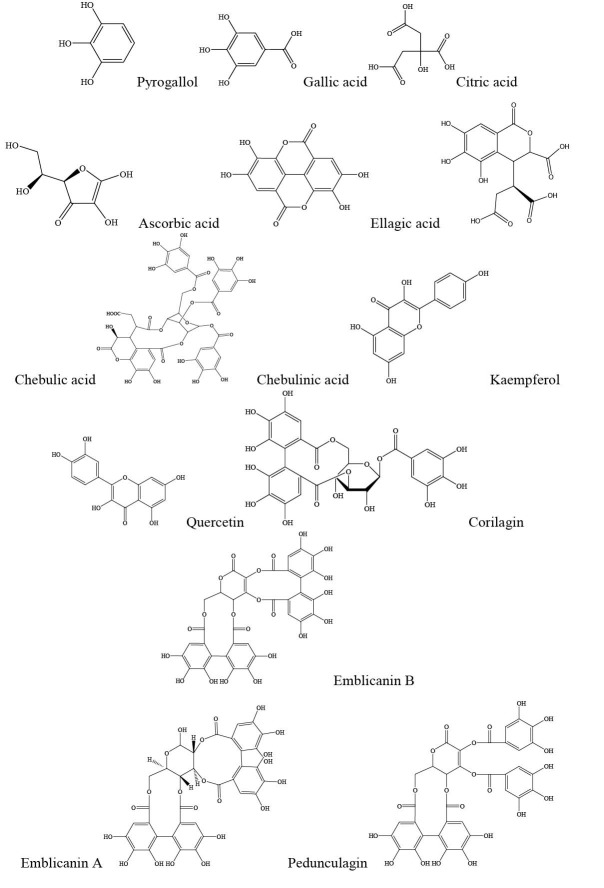
Structural formula of some important compounds of *Emblica officinalis.*

*Emblica officinalis* Gaertn. (Other names: *Phyllanthus emblica* Linn. and *Emblica pectinata* Ridl.) (family Euphorbiaceae), also known as Emblic myrobalan, Indian gooseberry, or amla, hereafter referred to as EO. This medium-sized deciduous tree is native to India and cultivated in Pakistan, Uzbekistan, Sri Lanka, South East Asia, China, and Malaysia.^7^ The EO fruits are used more than the other parts of the plant for treatment of various diseases in the Ayurveda and Unani medicines.^7^ The dried fruit is a common imported herbal product in the herbal markets of Iran. The fresh fruits resemble green sour plums about the size of a walnut (Figure 1). The fruit contains tannins, alkaloids, phenols, amino acids, carbohydrates, vitamins, flavonoids, and organic acids (Table 1 and Figure 2).^8,9^ The fruit is highly nutritious and consumed as a food.^10,11^

**Table 1:** Compounds of *Emblica officinalis*

**Class of compounds**	**Compounds**
Hydrolysable tannins	Emblicanin A and B, punigluconin, pedunculagin, chebulinic acid (ellagitannin), chebulagic acid (benzopyran tannin), corilagin (ellagitannin), geraniin (dehydroellagitannin), ellagotannin
Alkaloids	Phyllantine, phyllembein, phyllantidine
Phenols	Gallic acids, methyl gallate, ellagic acid, trigallayl glucose
Amino acids	Glutamic acid, proline, aspartic acid, alanine, cystine, lysine
Carbohydrates	Pectin
Vitamins	Ascorbic acid
Flavonoids	Quercetin, kaempferol
Organic acids	Citric acids

The EO fruit has also been mentioned in the literature of the Persian medicine (PM).^12^ EO is one of the 50 cardio-active plants mentioned in the Avicenna book “The Treatise on Cardiac Drugs”.^13^ Cardiotonic action is one of the features attributed to this plant in the PM.^12-14^ From the PM perspective, the fruit can be cardiotonic because it has astringent properties and can strengthen the cardiac tissue.^15^ Besides, it can affect the heart by exerting impact on stomach diseases (including gastro-esophageal reflux and mal-temperaments of the stomach, which are, as noted, related to cardiac diseases).^14^ Pharmacological studies have demonstrated diverse cardiovascular and other impacts for the fruit such as cytotoxic, hypoglycemic, hypolipidemic, hepatoprotective, cardioprotective, antiatherogenic, antioxidant, antipyretic, analgesic, antimicrobial, diuretic, and laxative effects.^7,16-18^

Numerous studies have been published on the effects of EO on various CVD; however, there has been no systematic review regarding the cardiovascular effects of EO nor is there a definitive decision on the efficacy of this plant. Therefore, this review was conducted to evaluate the plant potential for prevention and treatment of CVD.

## Materials and Methods

To collect the studies on the cardiovascular effects of EO, PubMed, Science Direct, Scopus, Proquest, Ebsco, Google, Google Scholar, Ovid, and Cochrane databases were searched for the English and non-English literature from 1966 to 2017 using the terms amla, Emblic myrobalan, *Emblica officinalis*, *Emblica pectinata*, Indian gooseberry, and *Phyllanthus emblica* together with antioxidant, arrhythmia, cardioprotective, cardiotoxicity, heart disease, heart failure, hyperlipidemia, hypertension, myocardial dysfunction, and oxidative stress. The database of Irandoc and the online libraries of Iranian universities were also searched for the purposes of this study.

Three persons performed the literature search and assessment. The inclusion criteria were in vitro, animal, and clinical cardiovascular pharmacological studies conducted on EO and full-text accessibility. The exclusion criterion was studies in which a combination of EO and at least one other plant was investigated. The reference lists of the retrieved articles were searched manually for additional eligible articles. All published cardiovascular pharmacological studies fulfilling the search criteria were included in the results section.

For clinical studies, PICO was considered as patients with a type of cardiovascular disease who took the EO with or without a control group with the aim to identify changes in CVD during intervention.

The methodological quality of clinical trials was assessed by the Jadad scale in terms of the presence of randomization, masking, and accountability of all patients including withdrawals, as described in the literature.^19^ The methodological quality of animal studies was assessed by ARRIVE checklist.^20^

**Figure 3:**
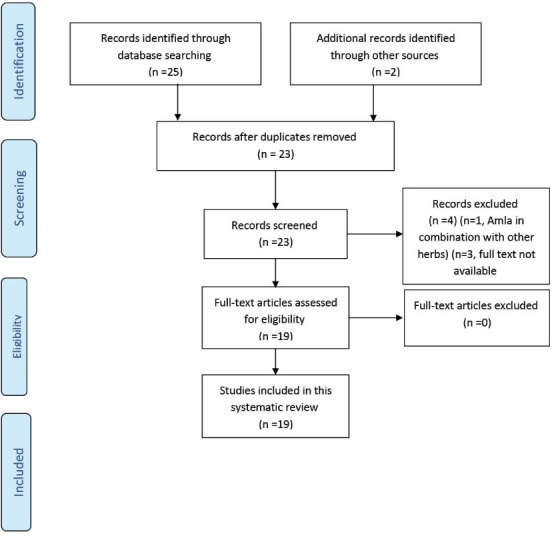
The PRISMA flow diagram of the study.

The PRISMA flow diagram of this review is presented in Figure 3.

## Results

There were no non-English and gray literature, theses and dissertations conforming to the search criteria. Summaries of the cardiovascular pharmacological studies fulfilling the search criteria are presented in Tables 2 and 3.

**Table 2:** Summary of the experimental pharmacological studies regarding the cardiovascular effects of *Emblica officinalis*

**Author, year**	**Methodological quality**	**Study design/participants/** **inclusion criteria**	**Intervention/control group**	**Outcome measure**	**Results**
Thakur CP. 1988^21^	High	N:100 (4 groups) cholesterol-induced hypercholesterolemic albino rabbits	Group1- control group (cholestrol 0.3 g/kg) Group 2- *Terminalia chebula* group (*Terminalia chebula*+ cholesterol) Group 3- *Terminalia belerica* group (*Terminalia belerica* + cholesterol) Group 4- the amla group (1 g/kg powdered EO + cholesterol, all interventions PO daily for 16 weeks	Serum TC, TG and LDL-C; cholesterol contents of the liver and aorta; fecal excretion of cholesterol	Decrease in serum TC and LDL-C, aortic and hepatic cholesterol, possibly through enzymatic degradation of cholesterol. No effect on fecal excretion of cholesterol. No effect on the serum TG.
Mathur R. 1996^22^	Medium	N: 28 (4 groups) cholesterol-induced hypercholesterolemic rabbits	Group 1-(control) vehicle treated for 60 and 120 days. Group 2- cholesterol feeding (400 mg/kg per day) for 60 and 120 days Group 3- cholesterol feeding for 60 days; afterward, cholesterol diet was withdrawn; control diet + fresh juice of EO from day 61 to day 120 (5 mL juice/kg per day). Group 4- cholesterol feeding + EO juice from day 1 to day 120 (concurrent feeding).	Serum TC, TG, LDL-C, VLDL-C and HDL-C; liver, ventricular muscle and aortic TC,TG and phospholipids contents.	Decrease in TC, TG and LDL-C levels. Decrease in the lipid levels of tissues. Regression of aortic plaques. Increase of fecal excretion of cholesterol and phospholipid.s.
Anila L. 2002^23^	High	N:30 (3 groups) cholesterol-induced hypercholesterolemic albino rats.	Group I - control Group II- flavonoids of EO10 mg/kg /day Group III - flavonoids of *Mangifera indica* PO for 90 days	Serum and liver LDL-C and VLDL-C and serum HDL-C. HMG-CoA activity.	Decrease of LDL-C and VLDL-C in serum and liver. Unchanged serum HDL-C. Inhibition of HMG-CoA and increased degradation and elimination of cholesterol.
Bhattacharya SK. 2002^24^	High	N:62 (8 groups) rat model of cardiac IRI	Group 1-saline perfusion (SP) for 30 minutes Group 2- tannoid principles of EO (EOT) (50 mg/kg) + SP Group 3-EOT (100 mg/kg) + SP Group 4-vitamin E (200mg/kg) + SP Group 5- IRI Group 6-EOT (50 mg/kg) + IRI Group 7-EOT (100 mg/kg) + IRI Group 8-vitamin E (200mg/kg) + IRI All perfused twice daily for 14 days	Cardiac SOD, catalase, glutathione peroxidase activity and lipid peroxidation	Both EOT (50 and 100 mg/kg) and vitamin E prevented IRI-induced effects (decrease in the activities of cardiac SOD, catalase and glutathione peroxidase, and increase in lipid peroxidation)
Rajak S. 2004^25^	Medium	N:64 rats (4 groups) rat model of cardiac IRI	Group1- normal rat Group 2- EO juice 250 mg/kg/day PO for 30 days Group 3- EO juice 500 mg/kg/day PO for 30 days Group 4- EO juice 750 mg/kg/day PO for 30 days	Myocardial TBARS (a measure of lipid peroxidation) content. Myocardial reduced glutathione, catalase, superoxide dismutase, glutathione peroxidase.	No significant increase in myocardial TBARS and depletion of antioxidant enzymes were observed after IRI in the treated groups. Myocyte injury was evident only in 250 mg/kg group.
Anthony B. 2006^26^	Medium	N:24 (4 groups) cholesterol-induced hypercholesterolemic rabbits.	Group 1- normal control Group 2- hypercholestrlemic control (vehicle PO) Group 3- Ethanol extract of EO 10 mg/kg/day PO Group 4- ethanol extract of EO 20 mg/kg/day PO for 4 months	Serum TC, TG, HDL-C, LDL-C; aortic atheromatous plaque; heart, liver and kidney cholesterol contents; HMG-CoA activity	Decrease of serum total cholesterol, TG and LDL-C; HDL-C increase; reduction of aortic atheromatous plaques; decrease of heart, liver and kidney cholesterol contents. Inhibition of HMG-CoA.
Patel SS. 2011^27^	Medium	N:48 rats (4 groups) rat model of diabetic-induced myocardial dysfunction	Group1- non-diabetic control Group 2-diabetic control Group 3-non-diabetic treated with EO juice 1 mL/kg/day PO for 8 weeks Group 4-diabetic treated with EO juice 1 mL/kg/day	Body weight, lipid profile, heart rate, BP, serum LDH and creatinine kinase-MB	EO prevented weight loss, hyperglycemia, dys-lipidemia, myocardial hypertrophy and cardiomyopathy; increased heart rate and force of contraction; Decreased BP and serum LDH and creatine kinase-MB in diabetic rats.
Bhatia J. 2011^28^	High	N:36 (6 groups) rat model of DOCA-salt-induced hypertension	Group 1- control group (vehicle) Group 2- DOCA 20 mg/kg SC plus 1% NaCl solution PO Groups 3–5- DOCA 20 mg/kg SC plus 1% NaCl solution and *E. officinalis* 75, 150 and 300 mg/kg/day PO, respectively Group 6: neither received DOCA 20 mg/kg SC nor 1% NaCl solution, received only *E. officinalis* 300 mg/kg/day PO plus normal drinking water.	SBP, DBP, mean arterial pressure, heart rate. Oxidative stress in serum, heart and kidney. Heart and kidney weights/100 g body weight ratio	Decrease of SBP, DBP, mean arterial pressure and heart rate. Increase of endothelial nitric oxide synthase activity and serum nitric oxide levels. Decrease of serum sodium and potassium levels. Decrease of oxidative stress in serum, heart and kidney. Decrease of renal and cardiac hypertrophy.
Ojha S. 2012^29^	High	N:40 (4 groups) rat model of isoproterenol-induced cardiotoxicity	Group 1- normal saline Group 2-Hydroalcoholic extract of EO 100 mg/kg PO Group 3- EO 250 mg/kg PO Group 4- EO 500 mg/kg PO for 30 days with concurrent isoproterenol (85 mg/kg SC) on 29^th^ and 30^th^ days.	Left ventricular pressure dynamics: peak positive pressure development, peak negative pressure decline and end diastolic pressure. Antioxidant enzymes, superoxide dismutase, catalase and glutathione peroxidase and myocyte-injury-specific marker enzymes creatine phosphokinase-MB and lactate dehydrogenase in heart	Restoration of hemodynamic and left ventricular function along with preservation of antioxidants, reduction of myocyte-injury-specific marker enzymes and inhibition of lipid peroxidation in EO groups.
Santoshkumar J. 2013^30^	High	N:30 (5 groups) cholesterol-induced hypercholesterolemic rats	Group 1- normal saline Group 2- powdered EO 540 mg/kg/day PO with normal diet Group 3- high fat diet Group 4- high fat diet and EO 540 mg/kg/day Group 5- high fat diet and atorvastatin 7.2 mg/kg/day, PO for 8 weeks	Serum TC,TG, LDL-C, HDL-C and atherogenic index	Decrease of TC, TG and LDL-C and atherogenic index and increase of HDL-C in EO and atorvastatin groups.
Rao TP. 2013^31^	Medium	Human umbilical vein endothelial cells. N: 40 rats (2 groups) LPS-induced endotoxemia rat model	In vitro application of EO water soluble extract (1-100 μ g/mL) on HUVEC in the presence of LPS. Group 1- control Group 2- Single dose of EO water soluble extract 50 mg/kg PO	LPS - induced tissue factor expression; von Willebrand factor level; LPS-induced adhesion of human monocytic cells (THP-1) to HUVEC; Expression of endothelial-leucocyte adhesion molecule-1 (E-selectin) in HUVEC. Pro-inflammatory cytokines TNF-α and IL-6 serum levels.	EO fruit extract reduced LPS - induced tissue factor expression and von Willebrand factor release in HUVEC and decreased LPS-induced adhesion of human monocytic cells (THP-1) to HUVEC and reduced expression of endothelial-leucocyte adhesion molecule-1 (E-selectin) in HUVEC. Reduction of TNF-α and IL-6.
Thirunavukkarasu M. 2015^32^	High	N:40 (4 groups) rat model of cardiac IRI	Group1- control sham Group 2- aqueous PE extract (100 mg/kg/day PO) for 30 days Group 3- control and IRI Group 4- PE and IRI	Western blot analysis and immunohistochemistry, phosphorylated Akt and GSK3-β, nitrotyrosine and caspase-3 expression, echocardiography	Preservation of myocardium during IRI through upregulation of PI3K/Akt/GSK3β/β-catenin. Increased ejection fraction and fractional shortening and decreased left ventricular internal diameter in electrocardiography of experimental subjects compared to controls.

N: sample size, EO: *Emblica officinalis* or Amla or PE: *Phyllanthus emblica*, TC: total cholesterol, TG: triglyceride, LDL: low density lipoprotein, HDL: high density lipoprotein, VLDL: very low density lipoprotein, SBP: systolic blood pressure, DBP: diastolic blood pressure, BMI: body mass index, hs-CRP: high-sensitivity C reactive protein, EOT: Emblica officinalis tannoids, IRI: Ischemia-reperfusion injury, SOD: superoxide dismutase, TBARS: thiobarbituric acid reactive substances, HUVEC: human umbilical vein endothelial cells, LPS: lipopolysaccharide, HMG-CoA: 3-hydroxy-3-methyl-glutaryl-coenzyme A , PO: orally, DOCA-salt: deoxycorticosterone acetate, LDH: Lactate dehydrogenase

**Table 3:** Summary of the clinical pharmacological studies regarding the cardiovascular effects of *Emblica officinalis*

**Author, year**	**JADAD score (out of 5)**	**Level of evidence/study design/** **participants/inclusion criteria**	**Intervention/control group**	**Outcome measure**	**Results**
Antony B. 2008^33^	<3	Level II quasi-experimental hypercholesterolemic (TC 190 – 310 mg/dL) patients.	Group1- n:22, aqueous extract of EO 500 mg o.d. PO for 6 months Group 2- n:17, aqueous extract of EO 1000 mg o.d. PO for 6 months	Serum TC, LDL-C, VLDL-C, TG, HDL-C and CRP	Reduction of TC, TG, LDL-C, VLDL-C and CRP and increase in HDL-C in both groups
Gopa B., 2012^34^	<3	Level II quasi-experimental N:60, type II hypercholestrolemic patients (TC>240 mg/dL)	Group 1- n:40, 500 mg amla capsule (dried amla fruit juice powder) o.d. PO for 42 days Group 2- n:20, simvastatin 20 mg o.d. PO for 42 days	Serum TC, TG, LDL-C,VLDL-C, HDL-C	Both treatments reduced TC, LDL-C,VLDL-C and TG and increased HDL-C
Gopa B, 2012^34^	<3	Level II quasi-experimental N:38, hypertensive patients (there is not further explanation)	Group 1- n:28, 500 mg amla capsule o.d. PO for 42 days Group 2- n:10, simvastatin 20 mg o.d. PO for 42 days	Systolic and diastolic blood pressure	21 patients on amla and 6 on simvastatin therapy showed improvement in blood pressure control
Usharani P 2013^35^	>3	Level I RCT N:80 type 2 diabetic patients HbA1c 7%-9%	Group1- PE extract 250 mg b.i.d. PO Group 2- PE extract 500 mg b.i.d. PO Group 3 -atorvastatin 10 mg o.d.PO + placebo Group 4 - placebo b.i.d. PO for 12 weeks	Change in endothelial function identified on salbutamol challenge, changes in serum malondialdehyde, nitric oxide, glutathione, hs-CRP, lipid profile and HbA1c	Reduction of endothelial reflection index in PE and atorvastatin groups, showing improvement of endothelial function possibly via anti-inflammatory and antioxidant actions. Reduction of serum total cholesterol, LDL-C, VLDL-C, TG, HbA1c, hs-CRP and MDA in PE and atorvastatin groups. Increased serum HDL-C, NO and glutathione in PE and atorvastatin groups.
Sinha RR et al 2014^36^	<3	Level I Randomized open label trial, type II hypercholestrolemic (TC>240 mg/dL and LDL-C>130 mg/dL) and hypertensive patients (there is not further explanation)	Group1 - n:45, 500 mg amla tablet (dried EO juice) b.i.d. PO for 16 weeks Group 2- n:48, atorvastatin 10 mg o.d. PO for 16 weeks	Serum TC, TG, HDL-C, LDL-C and VLDL-C levels, systolic and diastolic blood pressure	Amla was better in decreasing TG and increasing HDL-C, atrovastatin was better in decreasing TC, LDL-C and VLDL-C. BP did not significantly change.
Fatima N 2014^37^	>3	Level I crossover RCT N: 12 healthy participants	Aqueous extract of PE(250 mg) b.i.d. PO for 14 days Group 2- placebo capsule contains microcrystalline cellulose (49.7% w/w), lactose (49.5% w/w) and magnesium stearate (0.69% w/w) b.i.d. PO for 14 days	Heart rate, augmentation pressure, augmentation index (AIx), subendocardial viability ratio (SEVR), radial and aortic blood pressure were recorded before and after cold pressor test	PE extract decreased AIx, showing lowered systemic arterial stiffness. The extract reduced radial and aortic BP. It increased SEVR, showing increased myocardial oxygen supply/demand ratio.
Fatima N 2014^38^	>3	Level I Crossover RCT N:10 type 2 diabetic patients	Group1- 500 mg PE extract Group 2- 75 mg clopidogrel Group 3- 75 mg aspirin Group 4-500 mg PE + 75 mg clopidogrel Group 5- 500 mg PE + 75 mg aspirin, all as single dose. After single dose study and washout period, patients received Group 1-500 mg PE extract b.i.d. Group 2- 75 mg clopidogrel o.d. Group 3- 75 mg aspirin o.d. Group 4-combinations for 10 days. All treatments were given PO.	Platelet aggregation, bleeding time, clotting time	Decrease of platelet aggregation and increase of bleeding and clotting time compared to baseline in all groups
Khanna S. 2015^39^	<3	Level III quasi-experimental N:15 overweight/class-1 obese adults ( BMI: 25-35)	Aqueous extract of PE(500 mg) b.i.d. PO for 12 weeks	Serum hs-CRP level and platelet aggregation	Decrease in hs-CRP levels and downregulation of ADP- and collagen-induced platelet aggregation

RCT: randomized controlled trial, n: sample size, b.i.d.: two times daily, PO: orally. o.d.: once daily, BMI: body mass index, EO: *Emblica officinalis*, PE: *Phyllanthus emblica*, TC: total cholesterol, TG: triglyceride, LDL: low density lipoprotein, HDL: high density lipoprotein, VLDL: very low density lipoprotein, BP: blood pressure, , hs-CRP: high-sensitivity C reactive protein, BP: blood pressure, MDA: malondialdehyde, Aix: augmentation index, SEVR: subendocardial viability ratio, CPT: cold pressure test, NO: nitric oxide, ADP: Adenosine diphosphate.

The effects given in the results sections of the tables are statistically significant, unless otherwise stated.

## Discussion

This review collected and presented the evidences concerning the effects of EO on hyperlipidemia, hypertension, myocardial and endothelial function, cardiac specific antioxidants, and coagulation factors.

The results indicate that animal studies constitute the majority of EO-associated cardiovascular research. The plant affects a variety of cardiovascular parameters and has diverse cardiovascular pharmacological activities including antiatherogenic, anticoagulant, antidyslipidemic, antihypertensive, anti-inflammatory, antioxidant, antiplatelet, vasodilatory, and lipid deposition inhibitory effects. Moreover, it improves vascular endothelial function and prevents from both myocardial ischemia/reperfusion injury and doxorubicin and isoproterenol cardiotoxicity.

A few clinical studies have also been performed; nonetheless, the number of high quality clinical studies is not sufficiently large to provide a conclusive proposition on the efficacy of this plant in CVD.

The EO fruit is safe, and no side effects have been reported in clinical studies. In rats, the oral administration of the hydromethanolic (20:80) extract up to 2000 mg/kg over 15 days has not shown any clinical signs of toxicity.^40^ The animal studies demonstrated that the EO extract has anti-platelet activity and may increase the risk of bleeding when taken with drugs that increase the risk of bleeding. Some examples include aspirin, anticoagulants such as warfarin or heparin, anti-platelet drugs such as clopidogrel, and non-steroidal anti-inflammatory drugs (NSAIDS) such as ibuprofen or naproxen. *Phyllanthus emblica* extract interacts pharmacodynamically with clopidogrel and ecosprin in patients with type II diabetes mellitus.^38^

The EO fruit extract reduces blood sugar levels.^41,42^ Thus, caution should be taken when using hypoglycemic medications, and patients taking insulin or drugs for diabetes need to be monitored closely. In addition, EO decreases serum lipid levels; hence, cholesterol- or triglyceride-lowering medications should be taken with caution.

EO fruit is also a rich source of tannin and may interfere with intestinal absorption of iron.^43^

Since EO is a rich source of ascorbic acid, it may trigger gastric hyperacidity and constipation. Alongside this, in the PM literature, it is believed that EO has a cold and dry nature and may have an astringent property. To reduce the astringent effect of EO, it is advised to use as a jam or to consume it together with almond oil and honey.^12^ These must be considered in patients taking medications for their cardiovascular or other organs’ disorders.

Overall, it is concluded that EO affects various cardiovascular disorders and risk factors. However, there is not sufficient clinical evidence to suggest that EO has efficacy in CVD prevention and treatment. Further studies, especially clinical trials, with EO in all fields of cardiovascular pharmacology are needed. Identification of the bioactive compounds and mechanisms mediating the cardiovascular effects of EO is also suggestable.

## Competing interests

This review was not funded and there is no conflict of interest.

## Ethical approval

Not applicable.

## Acknowledgement

The authors thank Ms. Mahsima Abdoli for her cooperation in retrieval of the articles.
